# Differential host responses by the type I and III interferons in primary differentiated human nasal epithelial cells are a determinant of antiviral activity

**DOI:** 10.3389/fphar.2026.1895412

**Published:** 2026-09-15

**Authors:** Douglas Jie Wen Tay, Hui Yi Tay, Justin Jang Hann Chu, Kai Sen Tan

**Affiliations:** 1 Infectious Diseases Translational Research Programme and Department of Microbiology and Immunology, Yong Loo Lin School of Medicine, National University of Singapore, Singapore, Singapore; 2 NUSMed Biosafety Level 3 Core Facility, Yong Loo Lin School of Medicine, National University of Singapore, Singapore, Singapore; 3 Department of Medicine, Yong Loo Lin School of Medicine, National University of Singapore, Singapore, Singapore; 4 Singapore Lipidomics Incubator, Life Sciences Institute, National University of Singapore, Singapore, Singapore; 5 Laboratory of Molecular RNA Virology and Antiviral Strategies, Yong Loo Lin School of Medicine, National University of Singapore, Singapore, Singapore

**Keywords:** antiviral, human nasal epithelial cells, interferon transcriptome, type I interferon, type III interferon

## Abstract

**Background:**

The interferon (IFN) response is a crucial innate antiviral defence mechanism at the airway epithelium. The epithelial IFN response is predominantly mediated by the type I (IFN-β1) and III (IFN-λ) IFNs, which activate a transcriptional program expressing antiviral and inflammatory genes. Type III IFNs have been purported to be less inflammatory than type I IFN, but exhibit reduced potency. As both type I and III IFNs exert broad antiviral functions, there has been considerable interest in harnessing them as antiviral therapeutics. A better understanding of the effects of exogenous IFN on the host may enable its safer utilisation and prevent unwanted effects in clinical settings. Few studies have compared their potency using a standardised concentration or on primary human cells.

**Methods:**

Here, using differentiated primary human nasal epithelial cell (hNEC) cultures grown at the air-liquid interface, we compared the transcriptional profiles upon exogenous treatment (25 ng/mL) with IFN-β1 and IFN-λ1, -λ2, -λ3, and -λ4 across three time points (6 h, 24 h, and 72 h) by RNA-sequencing. Additionally, we investigated how the apicobasal polarity of the differentiated nasal epithelial influences IFN response by compartmentalised (apical vs. basal) treatment.

**Results:**

Our results revealed that the IFN response is weakly stimulated upon apical IFN treatment and requires additional basal treatment to mount an efficient antiviral response. Upon supplemental basal IFN treatment, IFN-β1 activates a robust transcriptional program of thousands of genes, of which the top differentially expressed genes are involved in the antiviral response and inflammatory pathways. The type III IFNs resulted in fewer genes (hundreds); amongst the type III IFNs, IFN-λ1 and -λ3 had comparable transcriptional profiles, followed by IFN-λ2. IFN-λ4 did not activate the IFN response at the tested concentration. Prophylactic treatment of hNECs with IFN-β1 or IFN-λ1 during influenza A infection demonstrated dose dependent antiviral properties, with IFN-β1 showing superior potency.

**Conclusion:**

Taken together, our study highlights the potency of type I IFN in antiviral control and provides a global IFN transcriptional profile in a physiologically relevant model that could serve as a fundamental framework of the type I and III IFN host response.

## 1 Introduction

Respiratory viruses are common causative agents in human diseases, leading to breathing difficulties, acute airway inflammation, and exacerbation of chronic airway inflammatory diseases (Tan et al., 2020; Eccles et al., 2023). The respiratory virus-host interaction begins at the nasal epithelium, the first site of contact for entry into the host (Wang et al., 2015). The nasal epithelium is a pseudostratified lining with apical (air-facing) and basolateral (extracellular matrix-facing) polarity and consists of three major cell types: (1) basal stem cells, which are involved in the renewal of the epithelium and differentiation into other cell types; (2) secretory cells, which secrete gel-forming mucus apically to trap inhaled pathogen and debris; and (3) ciliated columnar cells, which propel mucus out of the airway through coordinated cilia beating (Rock and Hogan, 2011; Yan et al., 2013; Bustamante-Marin and Ostrowski, 2017).

The interferon (IFN) antiviral response is an important component of the innate immune defence at the nasal epithelium, which is first initiated through the detection of pathogen-associated molecular patterns by pattern recognition receptors (Denney and Ho, 2018; Gallo et al., 2021). In the context of viral infection, viral RNA can be recognised by the Toll-like receptors within endosomes, and by the retinoic acid-inducible gene I in the cytosol. This results in a signalling cascade leading to the transcription of IFNs by IFN regulatory factor 3/7 and nuclear factor kappa light chain enhancer of activated B-cell (Akira et al., 2006; Iwanaszko and Kimmel, 2015). IFNs are a class of cytokines, named after their function to interfere with viral replication in the host, and classified into types I, II, and III (Negishi et al., 2018). The epithelial antiviral response is predominantly mediated by the type I and III IFNs (Lazear et al., 2019; Park and Iwasaki, 2020). Type I IFNs (mainly IFN-α and IFN-β1) signal through a heterodimeric receptor made up of IFN-α/β receptor alpha chains 1 and 2, found on the surface of all nucleated cells (Schreiber, 2017). In contrast, type III IFNs (consisting of IFN-λ1, -λ2, -λ3, and -λ4) signal through a different heterodimeric receptor made up of IFN-λ receptor 1 and IL10 receptor 2 that is mainly restricted to epithelial cells (Kotenko et al., 2003; Sheppard et al., 2003).

Both type I and III IFNs relay their signals through the Janus kinase-signal transducer and activator of transcription pathway to trigger the transcription of IFN-stimulated genes (ISGs) with broad-spectrum antiviral and inflammatory properties (Negishi et al., 2018). Despite signalling through a similar pathway, studies have reported that type I IFNs elicit a potent, rapid, and inflammatory response, whereas the type III IFN response is less potent, slow-acting, and less inflammatory (Casazza and Lazear, 2019; Forero et al., 2019; Lazear et al., 2019; Ye et al., 2019). There has been considerable interest in harnessing IFNs as antiviral therapeutics owing to their broad-spectrum activity (Chronopoulou and Tsochantaridis, 2025; Blumke and Seliger, 2026). Given the less inflammatory nature of type III IFNs compared to type I IFNs, it has been suggested that type III IFNs could be suitable antiviral treatments that exert minimal side effects on the host against respiratory viruses (Lazear et al., 2019). Additionally, the effect of type III IFNs is localised to epithelial cells which are the target sites for respiratory viruses and could prevent viral spread at the source. Indeed, therapeutic administration of recombinant IFN-λ in mouse models have demonstrated protection against respiratory viral infection and inhibition of pro-inflammatory cytokines (Davidson et al., 2016; Galani et al., 2017). However, studies in animal models may be confounded by species-specific responses that are different in human cells (Hermant et al., 2014; Jacobs et al., 2018). While datasets of IFN transcriptomic data are publicly available, there has been limited standardised comparisons of type I and III IFNs and their effect on human nasal epithelial cells (hNECs). To better utilise IFNs as an antiviral treatment at the upper airway epithelium, a systematic characterisation of the type I and III IFNs and their differential effect on the human epithelium is needed to understand the impact on the host.

The interrogation of host-pathogen interactions has been facilitated by the recent developments in cell culture models with a higher degree of physiological relevance compared to cell lines (Dapat and Oshitani, 2016; Hare et al., 2016). For example, the use of differentiated hNEC primary cultures grown at the air-liquid interface (ALI) have been shown to exhibit similar cell types, anatomical, and physiological processes as the *in vivo* nasal epithelium (Zhao et al., 2012). Under ALI conditions, the cells are differentiated with apicobasal polarity and polarised distribution of membrane receptor proteins, allowing the study of compartmentalised IFN stimulation that has been shown to affect the IFN response outcomes (Ciencewicki et al., 2009; Metz-Zumaran et al., 2024).

Here, we aim to compare the antiviral efficacy and characterise the temporal host transcriptional responses upon type I and III IFN stimulation at a standardised concentration using differentiated hNEC cultures. Our study provides a resource for the scientific community on the global IFN transcriptional profile in a physiologically relevant model that could serve as a fundamental framework of the type I and III IFN host and antiviral response.

## 2 Materials and methods

### 2.1 Primary human nasal epithelial stem/progenitor cell (hNESPC) culture and differentiation at air-liquid interface (ALI) to hNECs

Primary hNESPCs were purchased from Epithelix and expanded in DMEM/F12 (Gibco) supplemented with 10 ng/mL human epithelial growth factor (Gibco), 5 μg/mL insulin (Sigma-Aldrich), 0.1 nM cholera toxin (Sigma-Aldrich), 0.5 μg/mL hydrocortisone (Sigma-Aldrich), 2 nM 3,3′,5-triiodo-L-thyronine (Sigma-Aldrich), 1X N-2 supplement (Gibco), 1X antibiotic-antimycotic (Gibco), 1 μM A83−01 (Stemcell Technologies), 5 μM Y-27632 (Stemcell Technologies), and 3 μM isoprenaline hydrochloride (MedChemExpress) (Zhang et al., 2018; Tan et al., 2025). The expanded cells were seeded onto 24-well 0.4 μm polyester transwell inserts (Corning) and grown to confluence as submerged cultures (1–2 days). Once confluent, media from the apical and basal chambers were removed and 500 μL of PneumaCult-ALI medium with supplements (Stemcell Technologies) was added to the basal chamber to initiate differentiation at ALI. The basal medium was changed every 2–3 days and the cells were cultured at ALI for at least 28 days. Fully differentiated hNECs, characterised by observable cilia beating and mucus production, were used for the experiments. Approval for use of commercial primary human cells in this study was obtained from the National University of Singapore Institutional Review Board (NUS-IRB-2022-851).

### 2.2 Cell line

Madin-Darby Canine Kidney (MDCK; ATCC, CCL-34) cells were cultured in DMEM (Cytiva) and supplemented with 10% heat-inactivated FBS (Biowest). The cells were maintained at 37 °C with 5% CO_2_. Routine tests for mycoplasma were performed with the MycoAlert Mycoplasma Detection Kit (Lonza).

### 2.3 Influenza A virus

The H3N2 influenza A virus strain (A/Aichi/2/1968) was kindly provided by Dr Vincent Chow from the Department of Microbiology and Immunology, National University of Singapore.

### 2.4 Characterisation of hNEC ALI culture by immunofluorescence staining

Mature hNEC ALI cultures were fixed with 4% formaldehyde overnight and embedded in paraffin after a series of dehydration steps with the TP1020 Tissue Processor (Leica). The paraffin blocks were cut into 4 μm sections with the RM2255 microtome (Leica) and captured on poly-L-lysine-coated microscope slides (Epredia). The sectioned tissue was dewaxed and heated at 96 °C for 10 min in citrate buffer (Sigma-Aldrich) for antigen retrieval. Following permeabilization with 0.1% Triton X-100 and blocking with 10% normal goat serum (Invitrogen), the tissue was incubated with primary antibodies against acetylated α-tubulin (1:2,000, ab24610; abcam) and MUC5AC (1:400, ab198294; abcam) overnight at 4 °C. Incubation with fluorophore-conjugated secondary antibodies (1:500, Invitrogen, A-11029 and A-11037) was performed at room temperature for 1 h. The sections were mounted with Prolong Gold Antifade Mountant containing DAPI (Invitrogen) and imaged with the Mateo FL digital microscope (Leica) using the brightfield, blue (DAPI), green (acetylated α-tubulin), and orange (MUC5AC) channels. Composite image was prepared using ImageJ (MUC5AC was assigned to the red channel) with adjustment to the brightness and contrast.

### 2.5 IFN treatment of hNEC ALI cultures for RNA-sequencing

Recombinant human IFN-λ1 (1598-IL-025), λ2 (1587-IL-025), λ3 (5259-IL-025), λ4 (9165-IF-025; R&D Systems) and IFN-β1 (78113; Stemcell Technologies) were dissolved in Dulbecco’s phosphate buffered saline (DPBS) with 0.1% bovine serum albumin (BSA). The apical surface was washed with DPBS prior to treatment to remove excess mucus. The IFNs were diluted in DPBS with 0.1% BSA (for apical treatment) or PneumaCult-ALI medium with supplements (for basal treatment). For the untreated (vehicle) controls, only the diluent without IFNs was added to the cells. After 6 h, 24 h, and 72 h of treatment, the cells were lysed in 300 μL of mirVana lysis buffer (Invitrogen) for 2 min at room temperature to obtain cell lysates.

### 2.6 Transcriptomic analysis of IFN-treated hNEC ALI cultures

RNA from IFN-treated hNEC lysates was extracted with the mirVana miRNA Isolation Kit (Invitrogen) according to the manufacturer’s instructions for total RNA isolation. RNA was quantified by NanoDrop (Thermo Scientific) and at least 500 ng of RNA was submitted for RNA-sequencing by a commercial vendor (Azenta). Library preparation was performed on poly(A)-enriched RNA with the VAHTS Universal V8 RNA-seq Library Prep Kit for Illumina (Vazyme). Paired-end (150 bp) sequencing was performed on the Illumina NovaSeq system. Bioinformatic analyses were performed in-house. Briefly, FastQC (v0.12.1) was used to establish raw sequencing quality (Andrews, 2010). PolyG adapters were detected and trimmed using fastp (v0.23.4) (Chen et al., 2018). Alignment to the human reference genome (GRCh38. p14, Release 44) was performed using STAR (v2.7.11a) (Dobin et al., 2013). Gene expression levels (estimated counts and TPM) were estimated using RSEM (v1.3.3) (Li and Dewey, 2011). Qualimap (v2.3) and samtools (v1.15.1) were used as a quality control of the STAR alignment sequencing data (Garcia-Alcalde et al., 2012; Danecek et al., 2021). Differential gene expression (fold change to donor-matched untreated control) for each condition was assessed with DESeq2 (Love et al., 2014). Genes with an adjusted P-value of <0.05 were included in the analyses. Pathway analysis of significant genes was performed with the Metascape webtool (v3.5.20250701) (Zhou et al., 2019). The differentially expressed genes (DEGs) are provided in Supplementary Tables S1 and S2.

### 2.7 IFN pre-treatment and influenza A infection of hNEC ALI cultures

The hNEC ALI cultures were pre-treated with IFN or diluent (untreated controls) for 24 h prior to infection (25 ng/mL, 250 ng/mL or 1,000 ng/mL). Then, the apical and basal chambers were washed and replenished with fresh media in the basal chamber. Cultures were infected apically with influenza A (H3N2) at a MOI of 0.1 at 35 °C with 5% CO_2_ for 1 h (Hong et al., 2021; Tan et al., 2025). The inoculum was removed, and the cultures were incubated for 48 h at 35 °C with 5% CO_2_. After 48h, the apical chamber was incubated with 100 µL of DPBS for 10 min (apical wash) and collected for viral titration. Cells were lysed in 1X Laemmli sample buffer for the detection of viral antigen by Western blot.

### 2.8 Influenza A virus plaque assay

MDCK cells were seeded in 24-well plates overnight. Apical washes were serially diluted 10-fold in DMEM, added onto confluent MDCK cells, and incubated for 1 h at 35 °C with 5% CO_2_. Virus inoculum was removed after 1 h of adsorption, overlaid with 1.2% Avicel (International Flavors & Fragrances)-MEM (Cytiva) containing 0.5 μg/mL TPCK-trypsin (Sigma-Aldrich) and incubated for 3 days at 35 °C with 5% CO_2_. Cells were fixed with 4% formaldehyde for 1 h and stained with 0.5% crystal violet for 15 min. Viral titre was calculated with the formula:

Viral titre PFUml=Number of plaquesVolume of inoculum ml×Dilution factor.

### 2.9 Western blot

Western blot was performed to evaluate the intracellular levels of viral protein after pre-treatment of ALI cultures with increasing doses of IFN. Following 48 h of infection, cells were lysed in 1X Laemmli buffer. Proteins were electrophoresed in 10% SDS-PAGE gels and transferred onto 0.2 µm PVDF membranes with the Trans-Blot Turbo System (Bio-Rad). The membranes were blocked with 5% non-fat milk in Tris-buffered saline containing 0.1% Tween-20 (TBST) for 1 h at room temperature with rocking and incubated with primary antibodies (anti-H3N2 HA, 1:1,000, Invitrogen, PA5-34930 or anti-actin, 1:10,000, Millipore, MAB1501) at 4 °C overnight. The membranes were washed 5 times (5 min each) with TBST and incubated with the appropriate HRP-conjugated secondary antibody for 1 h at room temperature. After washing the membranes 5 times with TBST, the blots were detected with the Clarity Western ECL substrate (Bio-Rad) on the ChemiDoc Touch Imaging System (Bio-Rad). Protein expression levels were quantified using ImageJ.

### 2.10 Statistical analysis

Statistical analyses were performed with GraphPad Prism 8. The statistical tests for each experiment are indicated in the figure legends.

## 3 Results

### 3.1 IFN pre-treatment of hNECs at the dual apical-basal route but not the apical-only route inhibits influenza A virus replication at a low dose

Studies on the antiviral effects of IFNs commonly employ cell lines, which lack the physiological relevance and localisation of receptors. Thus, we first investigated the antiviral properties of the type I and type III IFNs on influenza A virus replication using differentiated primary hNEC ALI cultures. Differentiation of hNEC ALI cultures was confirmed by immunofluorescence staining of acetylated α-tubulin (marker of ciliated cells) and MUC5AC (marker of mucus-producing goblet cells) as shown in Figure 1A and Supplementary Figure S1. A low dose of 25 ng/mL was selected based on prior work by our group (using IFN-α 2A) (Hong et al., 2021). ALI cultures were pre-treated for 24 h with IFNs at both the apical and basal routes to mimic the bidirectional IFN release upon viral infection (Ciencewicki et al., 2009), or at the apical-only route to mimic the intranasal administration of exogenous IFN (Figure 1B). The cultures were then infected with influenza A virus (H3N2) apically for 48 h at an MOI of 0.1. When administered at both the apical and basal routes, IFN-β1 resulted in the greatest decrease (0.86-log_10_ reduction) in viral titre compared to the untreated control, while the type III IFNs had similar levels of reduction (0.077-log_10_ to 0.14-log_10_ reduction) apart from IFN-λ4 (Figure 1C). Interestingly, when administered at the apical-only route, the antiviral effects of all IFNs decreased as compared to administering at both the apical and basal routes (Figures 1D,E). These results suggest that at the same low dosage, the antiviral effect of type I IFN is more potent than the type III IFNs and that priming at the apical route results in inefficient antiviral activity.

**FIGURE 1:**
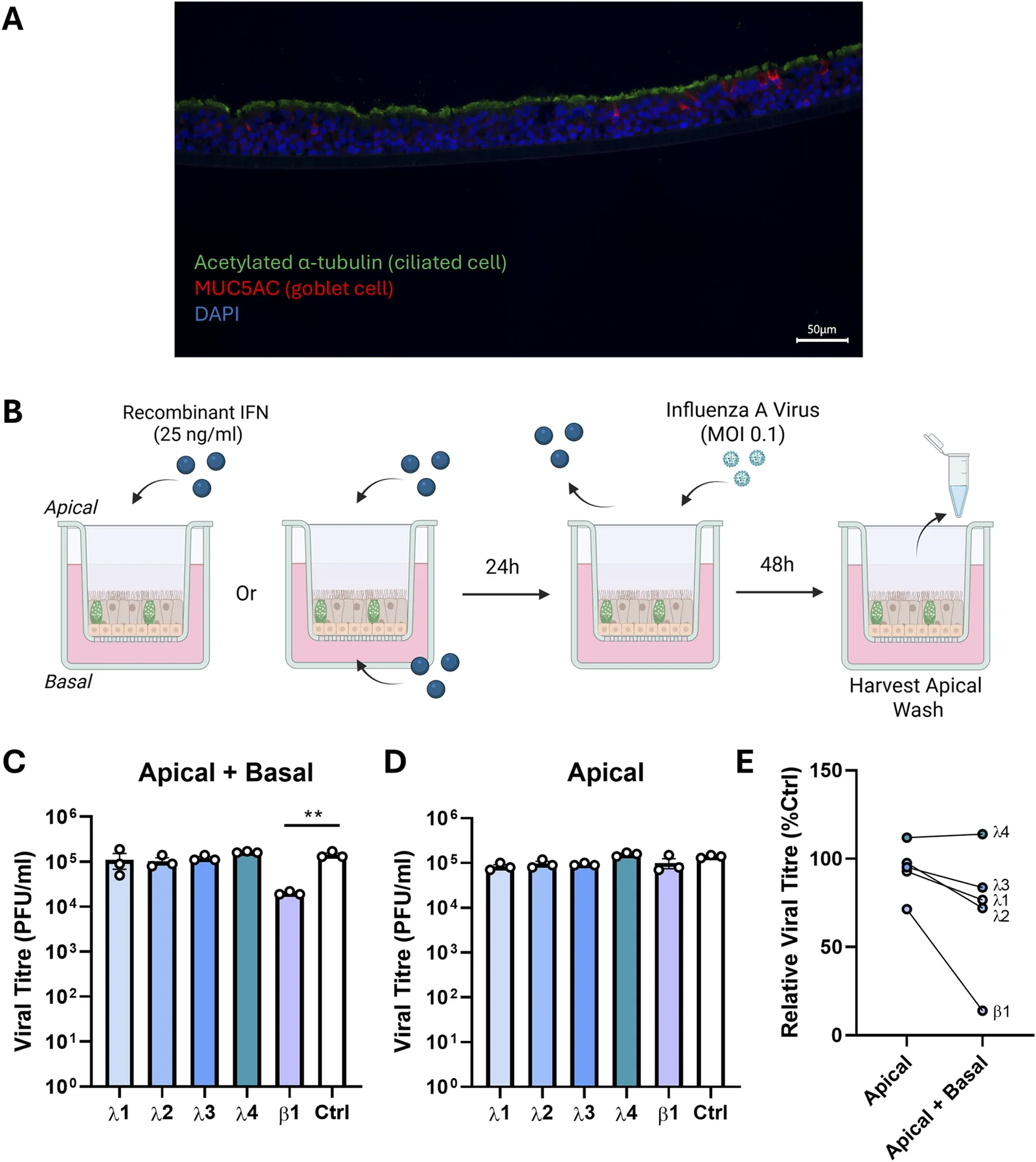
Interferon (IFN) pre-treatment of human nasal epithelial cells (hNECs) at the apical with basal route but not the apical-only route inhibits influenza A virus replication at a low dose. **(A)** Immunofluorescence image of the differentiated hNEC culture. Cross-section of ALI culture was stained for acetylated α-tubulin for ciliated cells (green) and MUC5AC for mucus-producing goblet cells (red). Images were taken with a ×20 objective lens. **(B)** Schematic illustration of IFN pre-treatment followed by influenza A virus infection. Differentiated hNEC cultures were individually pre-treated with 25 ng/mL of type I IFN (IFN-β1), type III IFN (IFN-λ1, IFN-λ2, IFN-λ3, or IFN-λ4) or with vehicle control for 24 h, followed by influenza A virus infection apically at a MOI of 0.1 for 48 h. The apical wash was collected to measure viral replication by plaque assay. **(C,D)** Virus titre in the apical wash of cultures pre-treated with IFN at both the apical and basal surfaces **(C)** or apical-only **(D)**. **(E)** Comparison of relative virus titre by route of IFN administration from data in **(C,D)**. For **(C,D)**, data is presented as mean ± SEM (n = 3); statistical significance was analysed by one-way ANOVA (compared to vehicle control) with Dunnett’s multiple comparisons test, **P < 0.01.

### 3.2 Interferon treatment at the dual apical-basal route results in robust gene induction while the apical-only route results in limited gene induction

The transcriptomic changes induced by IFN signalling results in the expression of ISGs, which contribute to the antiviral state of the host to limit viral infection and other host processes. To investigate the differences in antiviral activities between the type I and type III IFNs and the choice of treatment route on gene expression changes, we performed RNA-sequencing to assess the global transcriptome of IFN-treated hNEC ALI cultures obtained from five biological donors (Figure 2A). The hNEC ALI cultures were stimulated with type I or type III IFNs apically or both apically and basally for 6 h (early), 24 h (intermediate), or 72 h (late) and collected for RNA extraction at the indicated time points. Untreated controls were stimulated with the diluents without IFNs and served as the comparator (donor-matched) for the calculation of DEGs. In this study, we classified DEGs as genes with an adjusted P-value of less than 0.05 without an arbitrary fold change cut-off to account for weakly induced or repressed genes.

**FIGURE 2:**
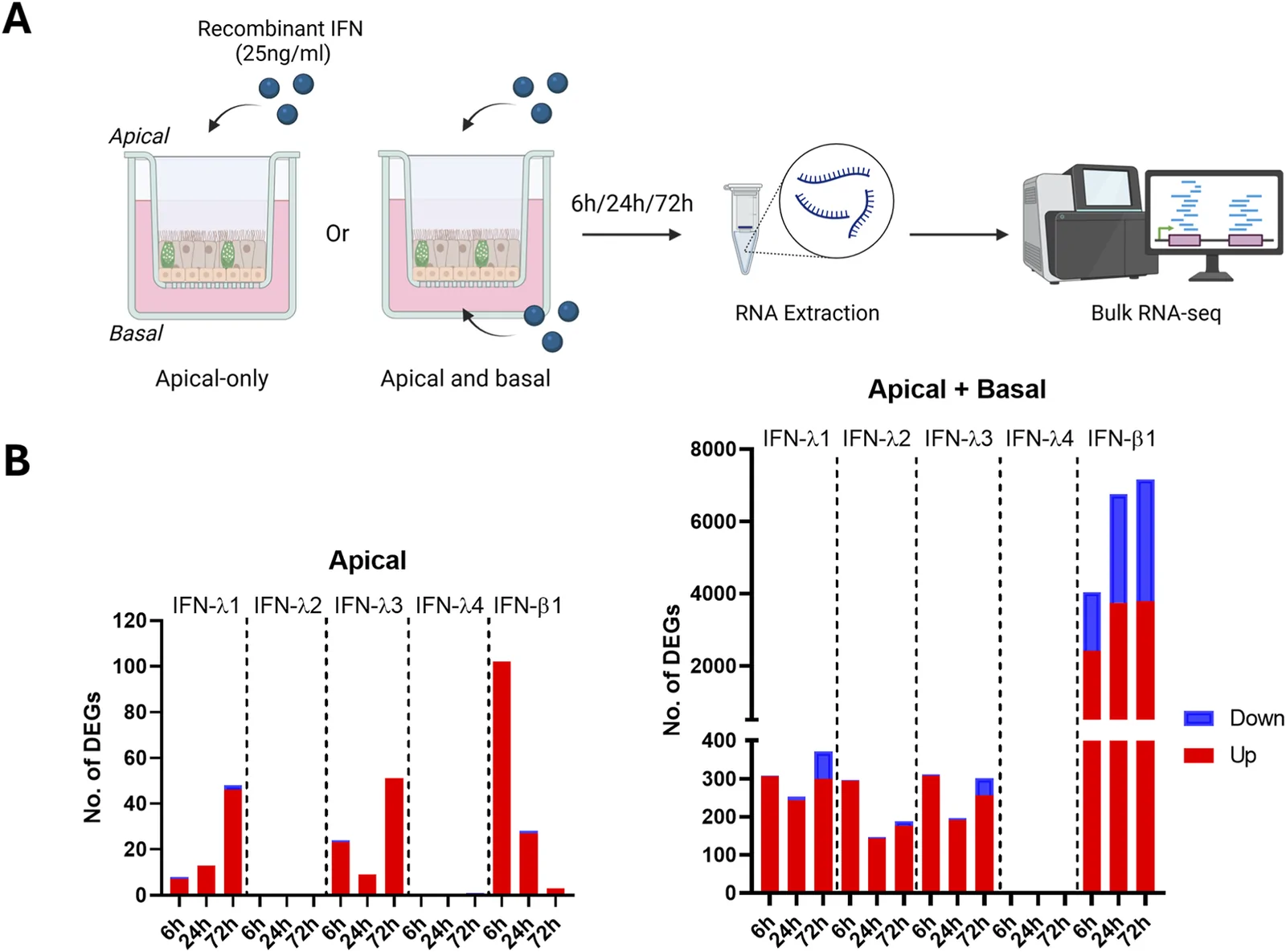
Interferon (IFN) treatment at the apical with basal route results in robust gene regulation while the apical-only route results in limited gene regulation. **(A)** Schematic illustration of IFN treatment for bulk RNA-sequencing. Differentiated human nasal epithelial cell cultures were individually pre-treated with 25 ng/mL of type I IFN (IFN-β1), type III IFN (IFN-λ1, IFN-λ2, IFN-λ3, or IFN-λ4) or with vehicle control for 6 h, 24 h, or 72 h. Cell lysates were collected for RNA extraction followed by bulk RNA-sequencing (n = 5). **(B)** Number of differentially expressed genes (DEGs) by route of treatment and IFN administered over time. DEGs were defined as genes with an adjusted P-value <0.05. Blue: downregulated genes, red: upregulated genes.

Treatment via the apical-only route resulted in the induction of few genes compared to treatment at both the apical and basal routes (Figure 2B). While IFN-λ2 did not result in transcriptomic changes when administered at the apical-only route, we observed similar levels of DEGs to those induced by other type III IFNs when administered at both the apical and basal routes. As our hNEC ALI cultures were not responsive to recombinant IFN-λ4, this treatment group was thus excluded from subsequent analyses.

At the apical-only route, type III IFNs were slow acting with the peak gene expression changes at 72 h, whereas type I IFN (IFN-β1) peaked early at 6 h and sharply decreased at 72 h (Figure 2B). In contrast, when administered at both the apical and basal routes, gene expression changes by type III IFNs started early at 6 h and sustained at comparable levels over time, with the lowest changes observed at 24h, whereas the type I IFN (IFN-β1) peaked at 24 h and maintained at similar levels until 72 h. In addition, the number of repressed genes increased over time. These observations highlight the differential gene induction kinetics based on the route of administration and suggest that treatment at both the apical and basal routes results in a more robust transcriptomic change compared to the apical-only route.

### 3.3 IFN treatment at the apical-only route results in limited induction of antiviral genes

We first performed pathway analysis of the DEGs from the apical-only IFN stimulation groups to identify changes in the host and antiviral response. At the early (6 h) time point, IFN-β1 stimulation resulted in the highest number of upregulated genes (102 genes), corresponding to the increased number of antiviral genes (GO:0051607 defence response to virus) compared to the type III IFNs (Figure 3A). There was a decrease of antiviral genes at the intermediate (24 h) time point for the IFN-β1 and IFN-λ3 groups, but a slight increase for the IFN-λ1 group (Figure 3B). At the late (72 h) time point, IFN-β1 stimulation resulted in the upregulation of only three genes (*IFI6, IFI44L*, and *IFI27*), which were all involved in the antiviral response. In contrast, there was an increase in the number of DEGs induced by type III IFNs, corresponding to an increase in antiviral genes (Figure 3C). Across the time points, the common pathways were related to antiviral defence (e.g., GO:0051607, GO:0045071, GO:0048525) and IFN response (e.g., GO:0034340, GO:0035455, GO:0035456). To visualise the number of common DEGs across the treatment conditions and time points, we generated an UpSet plot using the top five groups with the greatest number of DEGs (IFN-β1 6 h, IFN-β1 24 h, IFN-λ1 72 h, IFN-λ3 6 h, and IFN-λ3 72 h), which resulted in 31 intersect combinations (Supplementary Figure S2) (Lex et al., 2014; Gu, 2022). The common genes were mostly related to antiviral functions, such as the *IFI, IFIT, IFITM, MX*, and *OAS* family of genes. Given that the top enriched pathway of apical IFN stimulation was for the defence response to virus (GO:0051607) term, we generated a heatmap of the DEGs identified in this pathway to compare the gene expression profile across the groups. The peak antiviral gene induction for the type I and type III IFNs occurred at 6 h and 72 h post stimulation, respectively (Figure 3D). This may explain the weak antiviral activity in our initial pre-treatment assay where cells were stimulated for 24 h (Figure 1D), which had fewer upregulated antiviral genes (Figure 3D). We observed a modest reduction in viral titre with IFN-β1 pre-treatment, which may be contributed by the additional induction of *DDX60, DHX58, GBP4, HERC5, IFIT3, IFITM1, IFITM3, OAS1* and *OASL* that were not present in the type III IFNs treatment.

**FIGURE 3:**
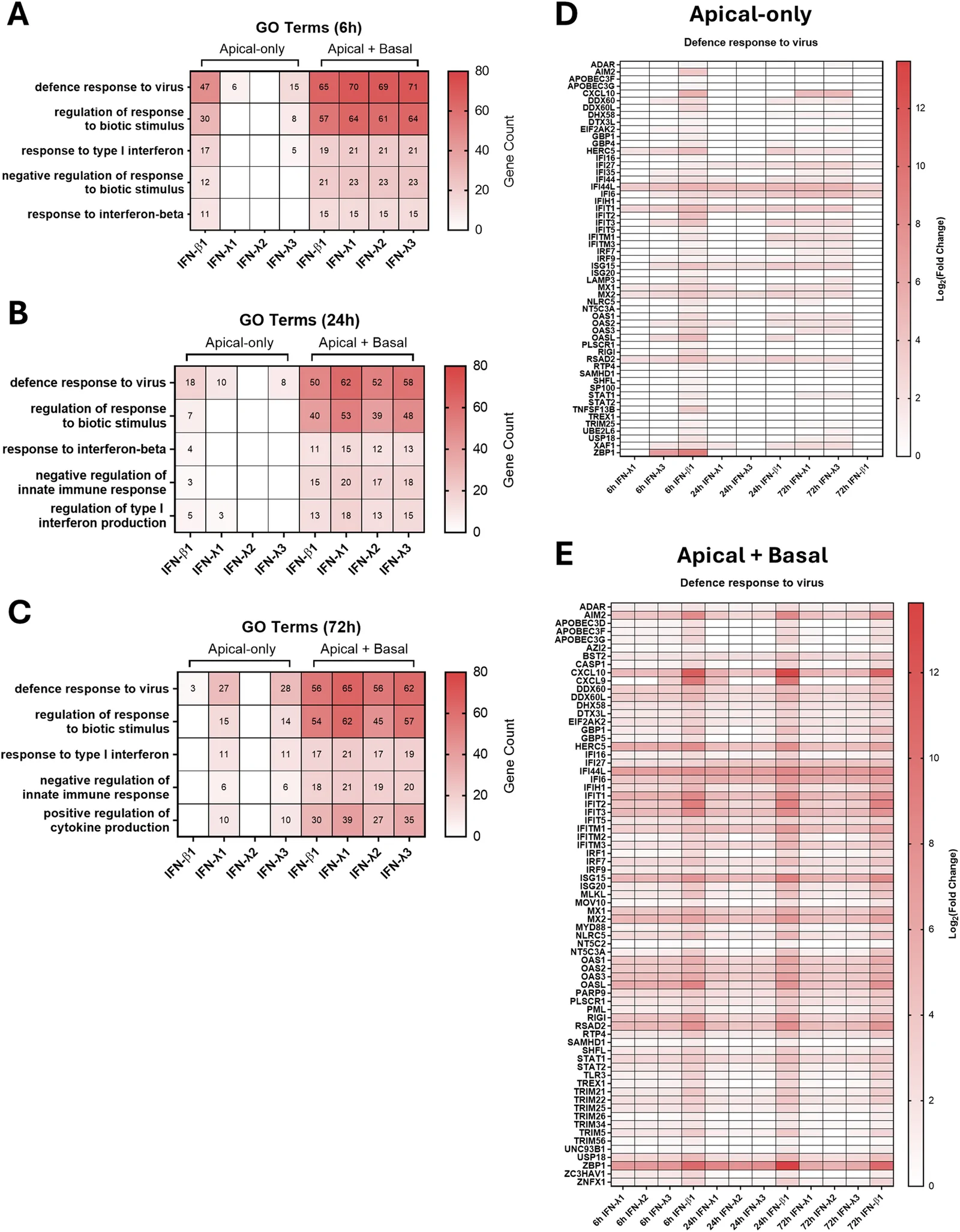
Interferon (IFN) treatment at the apical-only route results in limited induction of antiviral genes whereas the apical with basal route results in robust induction of antiviral genes. **(A–C)** Gene Ontology (GO) pathway analysis of upregulated differentially expressed genes (DEGs) from the apical-only or apical with basal treatment routes with IFN-β1, IFN-λ1, IFN-λ2, or IFN-λ3 at the 6 h **(A)**, 24 h **(B)**, and 72 h **(C)** time points. **(D,E)** Gene expression heatmap for DEGs in the “defence response to virus (GO:0051607)” GO term for the apical-only route **(D)** or the apical with basal route **(E)**.

### 3.4 IFN treatment at the dual apical-basal route results in robust induction of antiviral genes

We next performed pathway analysis of the upregulated DEGs from the dual apical and basal IFN stimulation groups. Due to the large number of DEGs with our cut-off for the IFN-β1 treatment group, we filtered the top ∼300 upregulated genes by the adjusted P-value followed by the fold change for inclusion in the pathway analysis. The top 5 GO terms across the IFNs at the different time points were also related to antiviral defence and IFN response, although at the intermediate (24 h) and late (72 h) time points, there was a negative regulation of the innate immune response (GO:0045824; Figures 3B,C). Compared to the apical-only IFN stimulation groups, there was a greater number of genes related to the defence response to virus (GO:0051607). From the gene expression heatmap, the fold changes of the DEGs were similar between the type III IFNs but were lower compared to the type I IFN-β1 (Figure 3E). The increased number and expression of antiviral DEGs may have contributed to the anti-influenza activity in the pre-treatment assay (Figure 1C), such as the *TRIM* gene family which was not induced in the apical-only route (Di Pietro et al., 2013; Choudhury et al., 2022; Lin et al., 2023).

### 3.5 IFN-β1 treatment at the dual apical-basal route additionally upregulates immune responses and downregulates transcriptional and translational processes

IFN-β1 stimulation at the dual apical-basal route resulted in substantial regulation of thousands of genes from 6 to 72 h. To further investigate the host responses caused by IFN-β1, we filtered out genes that were uniquely upregulated by IFN-β1 (Figure 4A). Next, we selected the top 300 genes (ranked by smallest adjusted P-value followed by largest fold increase) for GO pathway analysis. These unique genes were related to immune responses such as cytokine signalling, regulation of adaptive response, and inflammation (Figure 4B). The inflammatory response was the top ranked pathway over time, which includes genes involved in chemokine signalling (e.g., *CCR1, CCR3, CCL22, CCRL2, CXCL13, JAK2*) and inflammation (e.g., *GSDMB*; Figure 4C). We also analysed the top 300 downregulated genes (ranked by smallest adjusted P-value followed by largest fold decrease) and identified pathways related to host transcription and translation (Figure 4D). The downregulation of transcription and translation by type I IFNs have been reported as a mechanism to restrict viral replication processes (Ivashkiv and Donlin, 2014).

**FIGURE 4:**
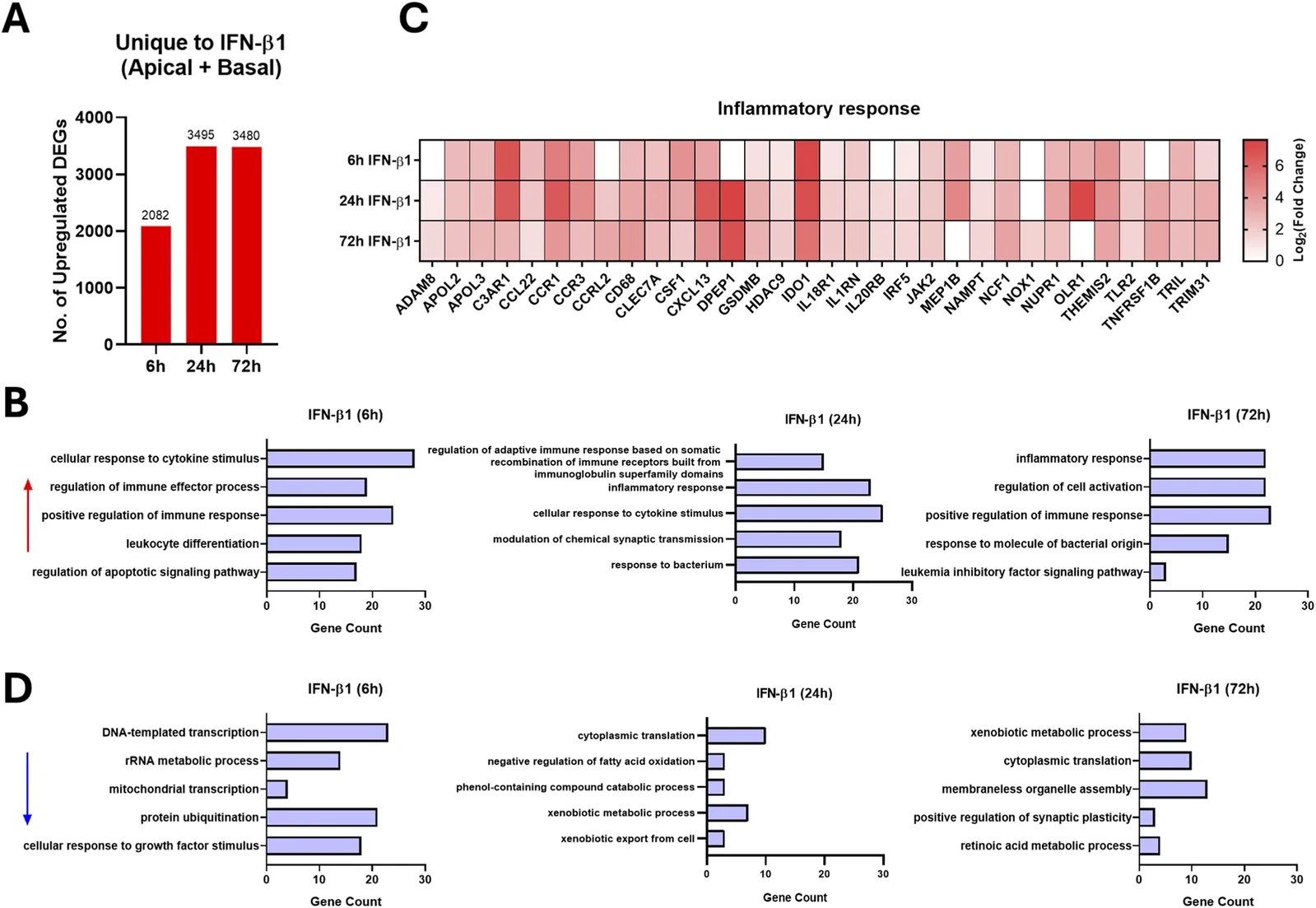
Interferon (IFN)-β1 treatment at the apical and basal route additionally upregulates immune responses and downregulates other processes. **(A)** Number of unique upregulated differentially expressed genes (DEGs) from the apical and basal treatment with IFN-β1 at the 6 h, 24 h, and 72 h time points. **(B)** Top Gene Ontology (GO) pathway terms from upregulated DEGs. **(C)** Gene expression heatmap for DEGs involved in inflammatory response. **(D)** Top GO pathway terms from downregulated DEGs.

### 3.6 The antiviral effect of IFNs is dose and route dependent

As the treatment with a low dosage (25 ng/mL) of IFN did not result in substantial antiviral gene response and reduction in viral titre, we hypothesised that increasing the IFN concentration could increase the potency of antiviral response. To this end, we pre-treated hNEC ALI cultures with 25 ng/mL, 250 ng/mL, or 1,000 ng/mL of IFN-β1 (type I IFN) or IFN-λ1 (representative type III IFN) for 24 h prior to influenza A infection. In our transcriptomic analyses, we observed that the combined apical and basal IFN stimulation resulted in a greater number of DEGs compared to the apical-only stimulation. Therefore, we included a basal-only stimulation condition as a comparator to evaluate its contribution to priming the host antiviral response. We measured intracellular viral HA protein production and infectious progeny in the apical wash 48 h post infection. In general, there was a dose dependent decrease in viral HA protein for IFN-β1 (Figures 5A,B) and virus titre (Figure 5C) with increasing concentration of IFN. Surprisingly, the basal-only route resulted in the greatest decrease in viral replication compared to the other treatment routes and was evident even at a low dose of 25 ng/mL. This decrease was not due to cellular toxicity caused by high dosage of IFNs (Supplementary Figure S3A). At the apical-only route, increasing the dosage of type I but not type III IFN was effective at reducing virus titre. Additionally, we assessed whether the increased dosage of IFN-λ1 would upregulate inflammatory response-related genes that were uniquely upregulated by IFN-β1 (Figure 4C). Of the selected genes, we observed minimal increase in expression with increasing dosage of IFN-λ1 compared to IFN-β1, suggesting the possibility of utilising higher dosage of IFN-λ1 for antiviral therapy (Supplementary Figure S3B). Taken together, these results suggest that the potency of the IFN antiviral response is dose and route dependent, with type I IFN eliciting a stronger effect compared to type III IFN.

**FIGURE 5:**
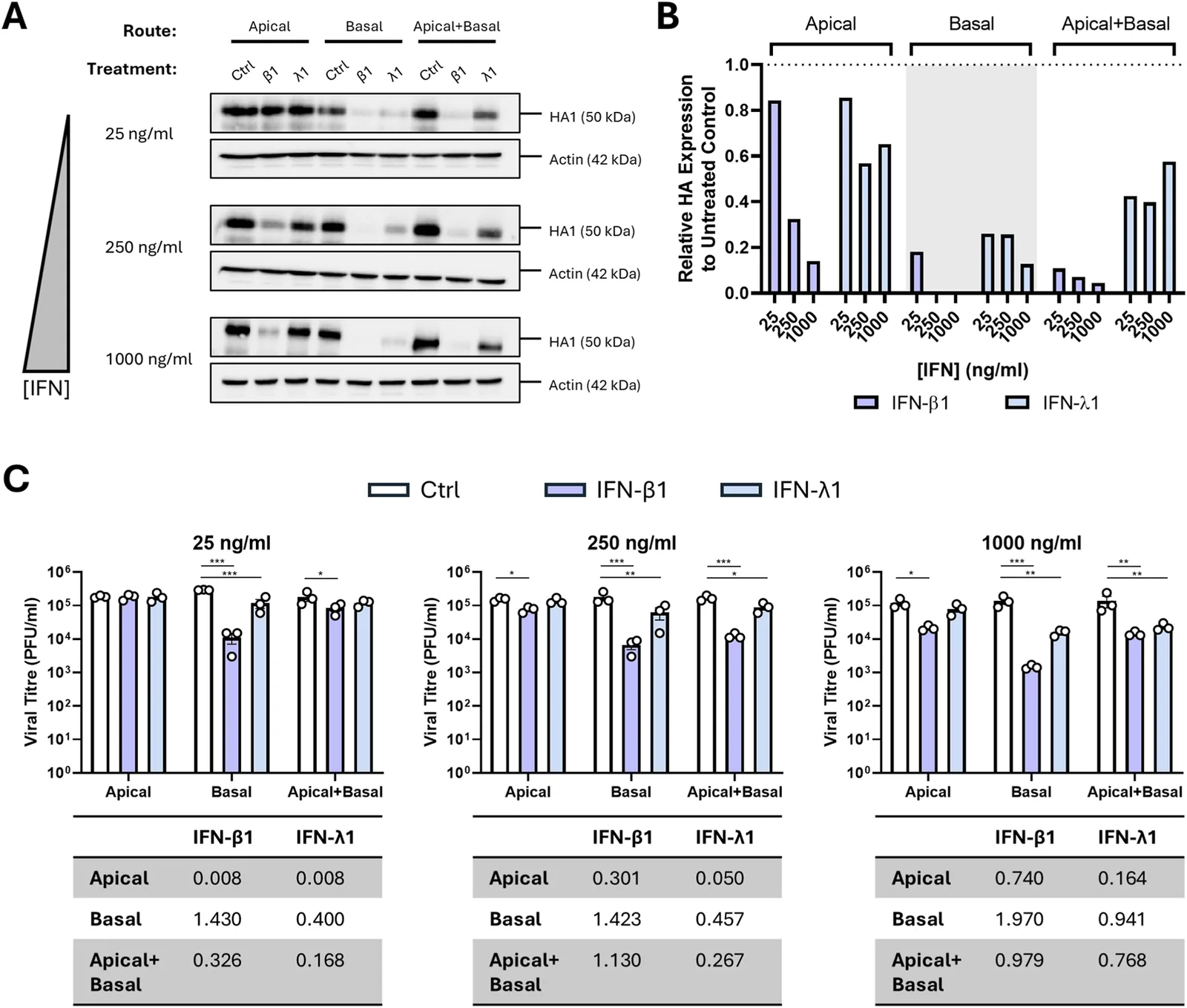
The antiviral effect of interferons (IFNs) is dose and route dependent. **(A–C)** Differentiated human nasal epithelial cell cultures were pre-treated with 25 ng/mL, 250 ng/mL or 1,000 ng/mL of IFN-β1, IFN-λ1, or with vehicle control for 24 h, followed by influenza A virus infection apically at a MOI of 0.1 for 48 h. The IFNs were administered apical-only, basal-only or at both apical and basal surfaces. **(A)** Western blot of intracellular influenza A haemagglutinin (HA). Actin served as the loading control (n = 1). **(B)** Densitometry values for **(A)**, relative to vehicle control (dotted line). **(C)** Virus titre in the apical wash. The log_10_ reduction in viral titre compared to vehicle control is presented in the tables under each plot. Data is presented as mean ± SEM (n = 3); statistical significance was analysed by two-way ANOVA (compared to vehicle control) with Dunnett’s multiple comparisons test, *P < 0.05, **P < 0.01, ***P < 0.001.

## 4 Discussion

In this study, we performed a standardised comparison to determine the antiviral efficacy and host transcriptional responses upon stimulation with type I and III IFN in a polarised hNEC model. Our initial pre-treatment experiment with 25 ng/mL of IFN showed that only IFN-β1 had an antiviral effect on influenza A virus. This antiviral effect was not prominent when IFN-β1 was only administered apically and required basal supplementation. RNA-sequencing of IFN stimulated hNEC cultures revealed that the apical-only IFN stimulation results in minimal induction of antiviral genes, which could have contributed to the lack of reduction in viral titre.

We observed that both IFN-λ2 and IFN-λ4 did not result in any DEGs in the apical-only route. However, IFN-λ2 induced comparable levels of DEGs as the other type III IFNs (IFN-λ1 and IFN-λ3) when administered both apically and basally, suggesting that the lack of effect apically was not due to an issue with the preparation. The potency of IFN-λ2 has generally been reported to be weaker than both IFN-λ1 and IFN-λ3, which may be attributed to its lower binding affinity for the receptor complex (Dellgren et al., 2009; Bolen et al., 2014; Keser et al., 2026). In addition, the IFN receptor is less abundant at the apical surface compared to the basolateral surface (Ciencewicki et al., 2009; Metz-Zumaran et al., 2024). We hypothesise that the weak receptor affinity of IFN-λ2 coupled with the sparse distribution of apical IFN receptors may be insufficient to initiate a positive feedback loop resulting in a transcriptional response. We also noted a lack of effect with IFN-λ4 in our study though a previous report showed the upregulation of a similar set of ISGs as IFN-λ3 in liver and airway epithelial cells with in-house recombinant IFN-λ4 preparations (Hamming et al., 2013). We postulate that the lack of effect with our commercial recombinant IFN-λ4 may be due to differences in bioactivity. Another explanation may be that IFN-λ4 acts transiently and its effect is not captured in our time points (Guo et al., 2021). Nevertheless, these observations highlight the differential activity of the type III IFN members when given apically, suggesting non-redundant roles and different capabilities in eliciting downstream transcriptional responses that warrants further investigation.

While type I IFN response is characterised as acute and rapid, with type III IFN response being prolonged and delayed in the literature, these kinetic differences in gene regulation were only observed in the apical-only treatment group in our study. We found that when IFN was administered at both the apical and basal surfaces, the number of genes regulated over time remained relatively stable. Due to the lower abundance of IFN receptors at the apical surface compared to the basal surface, there may be a weaker stimulation and subsequent signalling by exogenous IFNs apically (Ciencewicki et al., 2009). Additionally, ISGs differ in their transcriptional response and sensitivity by the IFN-stimulated response element (ISRE) motif found in the promoter sites (Van Winkle et al., 2020). Thus, we hypothesise that the weak apical stimulation by type I IFN preferentially enhances negative regulators with strong ISRE, such as *USP18*, which results in the characteristic decline in DEGs over time (Kang and Jeon, 2020; Van Winkle et al., 2020; Sun et al., 2025). We did not observe the induction of negative regulators of type III IFNs (e.g., *SOCS1*) in the type III IFN apical-only treatment groups, which could have allowed continual signalling and increase in DEGs over time. The minimal stimulation of apical IFN receptors may serve as a regulatory mechanism to prevent excessive immune response and tissue damage at the mucosal barrier (Humlicek et al., 2007). However, the activation of basolateral IFN receptors, typically caused by the release of IFNs by immune cells in the lamina propria, suggests a breach in epithelial integrity by pathogens that results in a robust transcription of ISGs (Ciencewicki et al., 2009). This may explain the sustained levels of DEGs over time in the dual apical-basal IFN treatment groups, which is potentially activated with the goal of pathogen clearance. These findings highlight the complexity of IFN signalling and suggest that IFN responses are spatially regulated. The use of polarised cell models is thus recommended to dissect the molecular intricacies of IFN response outcomes.

Several studies have explored the IFN transcriptomic signature using airway epithelial ALI cultures, though with differing time points (usually a single time point) and concentrations used (Lauber et al., 2015; Erb et al., 2022). A common finding from these studies is the observation that type I IFNs exert greater potency compared to type III IFNs. This could be due to differences in receptor binding affinity, receptor abundance, signal amplification and regulation (Mesev et al., 2019). From our RNA-sequencing data, we extracted the gene expression of type I IFN receptors (*IFNAR1* and *IFNAR2*) and type III IFN receptors (*IFNLR1* and *IL10RB*) from untreated control groups and found that *IFNLR1* had the lowest baseline expression amongst the receptor subunits (Supplementary Figure S4). The reduced abundance of a type III IFN receptor subunit may in part contribute to the reduced potency of type III IFN signalling. To provide a greater characterisation of the IFN response, we investigated multiple time points and compared a representative type I IFN (IFN-β1) with all the type III IFN subtypes, providing insights into the kinetics of gene induction by the different IFNs. Importantly, we investigated the differences in IFN responses resulting from compartmental administration, revealing the potency of basal stimulation over apical stimulation.

For the application of exogenous IFNs as an antiviral therapeutic, it would be critical to consider the route of administration to reach and activate basolateral IFN receptors to prime the host antiviral state. While this can be achieved by systemic delivery of IFNs, there may be unwanted side effects, such as an overt inflammatory response, especially with type I IFNs that act on all nucleated cells. Systemic delivery of type III IFNs may provide a more targeted therapeutic approach than type I IFNs as they act mainly on mucosal epithelial cells. However, the dosage and duration of systemic type III IFN administration need to be carefully considered as they may still disrupt the epithelial barrier with prolonged treatment (Broggi et al., 2020). In our study, we observed that increasing the dosage of IFN-λ1 resulted in minimal upregulation of a selected set of inflammatory response-related genes (e.g., *GSDMB, CXCL13*) that were highly upregulated by IFN-β1 even at low concentrations (Supplementary Figure S3B). This suggests the possibility of using a high dosage of IFN-λ1 to achieve antiviral efficacy matching the type I IFNs without its detrimental inflammatory effects. However, additional studies are required to evaluate the transcriptome-wide host changes upon stimulation with a high dosage of IFN-λ1. The sublingual administration route has been proposed to reduce IFN-related toxicity while achieving antiviral efficacies, though additional clinical studies are required to determine the effectiveness of this administration route (Beilharz et al., 2010; Arico et al., 2020). For local administrations, such as via nasal sprays, formulations of IFNs may be modified to favour transcytosis (e.g., by FcRn transporter) (Fieux et al., 2021).

This study has certain limitations. First, we utilised only one concentration of IFN for RNA-sequencing. Therefore, it remains unknown whether and how higher concentrations of IFN could negatively affect the host even if a strong antiviral effect is established. However, we have partially showed that even increasing doses of IFN-λ1 do not induce inflammatory response gene expression as strongly as IFN-β1 via selected RT-qPCR, suggesting milder adverse effect to the epithelium by type III IFNs. Second, we did not have a basolateral-only treatment group for RNA-sequencing. However, we included this treatment group in our pre-treatment experiment which demonstrated superior antiviral activity over the other routes of administration. Third, the recombinant IFNs were purchased from different vendors and may vary in terms of bioactivity.

Future work could utilise spatial omics to identify potential feedback mechanisms to account for the observed superiority of basal IFN treatment over the dual apical-basal IFN treatment group. Follow-up experiments could investigate whether there is an optimal duration for IFN treatment to achieve antiviral activity via the apical-only route (e.g., 6 h for type I IFNs and 72 h for type III IFNs). Additionally, co-culture models with immune cells in the basal chamber could provide a greater overall understanding of the airway epithelial IFN response with the inclusion of epithelial-immune cell interactions. Humanised IFN mouse models could provide physiological insights on the potential local or systemic pathological effects caused by IFN-induced inflammation to facilitate the evaluation for clinical use (Wang et al., 2023).

## 5 Conclusion

In conclusion, our study highlights the potency of type I IFN and the importance of basolateral stimulation with IFNs for antiviral control. These results contribute to the knowledge base on type I and III IFN host response using a physiologically relevant model, which could guide future studies to decipher the complexity of IFN response outcomes and optimise its use as an antiviral therapeutic.

## Data Availability

The datasets presented in this study can be found in online repositories. The names of the repository/repositories and accession number(s) can be found below: https://www.ncbi.nlm.nih.gov/geo/, GSE342661.

## References

[B1] Akira S. Uematsu S. Takeuchi O. (2006). Pathogen recognition and innate immunity. Cell 124 (4), 783–801. 10.1016/j.cell.2006.02.015 16497588

[B2] Andrews S. (2010). FastQC: a quality control tool for high throughput sequence data. Available online at: http://www.bioinformatics.babraham.ac.uk/projects/fastqc/ (Accessed December 21, 2023).

[B3] Arico E. Bracci L. Castiello L. Gessani S. Belardelli F. (2020). Are we fully exploiting type I interferons in today's fight against COVID-19 pandemic? Cytokine Growth Factor Rev. 54, 43–50. 10.1016/j.cytogfr.2020.07.010 32665127 PMC7334951

[B4] Beilharz M. W. Cummins M. J. Bennett A. L. Cummins J. M. (2010). Oromucosal administration of interferon to humans. Pharm. (Basel) 3 (2), 323–344. 10.3390/ph3020323 27713254 PMC4033912

[B5] Blumke J. Seliger B. (2026). Interferon lambda in anti-viral defense and cancer: dual roles, mechanism and therapeutic potential. J. Transl. Med. 24, 830. 10.1186/s12967-026-08209-8 42092929 PMC13317279

[B6] Bolen C. R. Ding S. Robek M. D. Kleinstein S. H. (2014). Dynamic expression profiling of type I and type III interferon-stimulated hepatocytes reveals a stable hierarchy of gene expression. Hepatology 59 (4), 1262–1272. 10.1002/hep.26657 23929627 PMC3938553

[B7] Broggi A. Ghosh S. Sposito B. Spreafico R. Balzarini F. Lo Cascio A. (2020). Type III interferons disrupt the lung epithelial barrier upon viral recognition. Science 369 (6504), 706–712. 10.1126/science.abc3545 32527925 PMC7292499

[B8] Bustamante-Marin X. M. Ostrowski L. E. (2017). Cilia and mucociliary clearance. Cold Spring Harb. Perspect. Biol. 9 (4), a028241. 10.1101/cshperspect.a028241 27864314 PMC5378048

[B9] Casazza R. L. Lazear H. M. (2019). Why is IFN-Lambda less inflammatory? One IRF decides. Immunity 51 (3), 415–417. 10.1016/j.immuni.2019.08.019 31533051

[B10] Chen S. Zhou Y. Chen Y. Gu J. (2018). Fastp: an ultra-fast all-in-one FASTQ preprocessor. Bioinformatics 34 (17), i884–i890. 10.1093/bioinformatics/bty560 30423086 PMC6129281

[B11] Choudhury N. R. Trus I. Heikel G. Wolczyk M. Szymanski J. Bolembach A. (2022). TRIM25 inhibits influenza A virus infection, destabilizes viral mRNA, but is redundant for activating the RIG-I pathway. Nucleic Acids Res. 50 (12), 7097–7114. 10.1093/nar/gkac512 35736141 PMC9262604

[B12] Chronopoulou S. Tsochantaridis I. (2025). Interferon lambda: the next frontier in antiviral therapy? Pharm. (Basel) 18 (6), 785. 10.3390/ph18060785 40573183 PMC12195726

[B13] Ciencewicki J. M. Brighton L. E. Jaspers I. (2009). Localization of type I interferon receptor limits interferon-induced TLR3 in epithelial cells. J. Interferon Cytokine Res. 29 (5), 289–297. 10.1089/jir.2008.0075 19231996 PMC2956593

[B14] Danecek P. Bonfield J. K. Liddle J. Marshall J. Ohan V. Pollard M. O. (2021). Twelve years of SAMtools and BCFtools. Gigascience 10 (2), giab008. 10.1093/gigascience/giab008 33590861 PMC7931819

[B15] Dapat C. Oshitani H. (2016). Novel insights into human respiratory syncytial virus-host factor interactions through integrated proteomics and transcriptomics analysis. Expert Rev. Anti Infect. Ther. 14 (3), 285–297. 10.1586/14787210.2016.1141676 26760927 PMC4819838

[B16] Davidson S. McCabe T. M. Crotta S. Gad H. H. Hessel E. M. Beinke S. (2016). IFNlambda is a potent anti-influenza therapeutic without the inflammatory side effects of IFNalpha treatment. EMBO Mol. Med. 8 (9), 1099–1112. 10.15252/emmm.201606413 27520969 PMC5009813

[B17] Dellgren C. Gad H. H. Hamming O. J. Melchjorsen J. Hartmann R. (2009). Human interferon-lambda3 is a potent member of the type III interferon family. Genes. Immun. 10 (2), 125–131. 10.1038/gene.2008.87 18987645

[B18] Denney L. Ho L. P. (2018). The role of respiratory epithelium in host defence against influenza virus infection. Biomed. J. 41 (4), 218–233. 10.1016/j.bj.2018.08.004 30348265 PMC6197993

[B19] Di Pietro A. Kajaste-Rudnitski A. Oteiza A. Nicora L. Towers G. J. Mechti N. (2013). TRIM22 inhibits influenza A virus infection by targeting the viral nucleoprotein for degradation. J. Virol. 87 (8), 4523–4533. 10.1128/JVI.02548-12 23408607 PMC3624352

[B20] Dobin A. Davis C. A. Schlesinger F. Drenkow J. Zaleski C. Jha S. (2013). STAR: ultrafast universal RNA-seq aligner. Bioinformatics 29 (1), 15–21. 10.1093/bioinformatics/bts635 23104886 PMC3530905

[B64] Eccles R. (2023). Common cold. Front. Allergy 4, 1224988. 10.3389/falgy.2023.1224988 37426629 PMC10324571

[B21] Erb A. Zissler U. M. Oelsner M. Chaker A. M. Schmidt-Weber C. B. Jakwerth C. A. (2022). Genome-wide gene expression analysis reveals unique genes signatures of epithelial reorganization in primary airway epithelium induced by Type-I, -II and -III interferons. Biosens. (Basel) 12 (11), 929. 10.3390/bios12110929 36354438 PMC9688329

[B22] Fieux M. Le Quellec S. Bartier S. Coste A. Louis B. Giroudon C. (2021). FcRn as a transporter for nasal delivery of biologics: a systematic review. Int. J. Mol. Sci. 22 (12), 6475. 10.3390/ijms22126475 34204226 PMC8234196

[B23] Forero A. Ozarkar S. Li H. Lee C. H. Hemann E. A. Nadjsombati M. S. (2019). Differential activation of the transcription factor IRF1 underlies the distinct immune responses elicited by type I and type III interferons. Immunity 51 (3), 451–464 e456. 10.1016/j.immuni.2019.07.007 31471108 PMC7447158

[B24] Galani I. E. Triantafyllia V. Eleminiadou E. E. Koltsida O. Stavropoulos A. Manioudaki M. (2017). Interferon-lambda mediates non-redundant front-line antiviral protection against influenza virus infection without compromising host fitness. Immunity 46 (5), 875–890 e876. 10.1016/j.immuni.2017.04.025 28514692

[B25] Gallo O. Locatello L. G. Mazzoni A. Novelli L. Annunziato F. (2021). The central role of the nasal microenvironment in the transmission, modulation, and clinical progression of SARS-CoV-2 infection. Mucosal Immunol. 14 (2), 305–316. 10.1038/s41385-020-00359-2 33244161 PMC7690066

[B26] Garcia-Alcalde F. Okonechnikov K. Carbonell J. Cruz L. M. Gotz S. Tarazona S. (2012). Qualimap: evaluating next-generation sequencing alignment data. Bioinformatics 28 (20), 2678–2679. 10.1093/bioinformatics/bts503 22914218

[B27] Gu Z. (2022). Complex heatmap visualization. Imeta 1 (3), e43. 10.1002/imt2.43 38868715 PMC10989952

[B28] Guo C. Reuss D. Coey J. D. Sukumar S. Lang B. McLauchlan J. (2021). Conserved induction of distinct antiviral signalling kinetics by primate interferon lambda 4 proteins. Front. Immunol. 12, 772588. 10.3389/fimmu.2021.772588 34868037 PMC8636442

[B29] Hamming O. J. Terczynska-Dyla E. Vieyres G. Dijkman R. Jorgensen S. E. Akhtar H. (2013). Interferon lambda 4 signals via the IFNlambda receptor to regulate antiviral activity against HCV and coronaviruses. EMBO J. 32 (23), 3055–3065. 10.1038/emboj.2013.232 24169568 PMC3844954

[B30] Hare D. Collins S. Cuddington B. Mossman K. (2016). The importance of physiologically relevant cell lines for studying virus-host interactions. Viruses 8 (11), 297. 10.3390/v8110297 27809273 PMC5127011

[B31] Hermant P. Demarez C. Mahlakoiv T. Staeheli P. Meuleman P. Michiels T. (2014). Human but not mouse hepatocytes respond to interferon-lambda *in vivo* . PLoS One 9 (1), e87906. 10.1371/journal.pone.0087906 24498220 PMC3909289

[B32] Hong H. Tan K. S. Yan Y. Chen F. Ong H. H. Oo Y. (2021). Induction of IL-25 expression in human nasal polyp epithelium by influenza virus infection is abated by interferon-alpha pretreatment. J. Inflamm. Res. 14, 2769–2780. 10.2147/JIR.S304320 34234504 PMC8254189

[B33] Humlicek A. L. Manzel L. J. Chin C. L. Shi L. Excoffon K. J. Winter M. C. (2007). Paracellular permeability restricts airway epithelial responses to selectively allow activation by mediators at the basolateral surface. J. Immunol. 178 (10), 6395–6403. 10.4049/jimmunol.178.10.6395 17475869

[B34] Ivashkiv L. B. Donlin L. T. (2014). Regulation of type I interferon responses. Nat. Rev. Immunol. 14 (1), 36–49. 10.1038/nri3581 24362405 PMC4084561

[B35] Iwanaszko M. Kimmel M. (2015). NF-kappaB and IRF pathways: cross-regulation on target genes promoter level. BMC Genomics 16 (1), 307. 10.1186/s12864-015-1511-7 25888367 PMC4430024

[B36] Jacobs S. Wavreil F. Schepens B. Gad H. H. Hartmann R. Rocha-Pereira J. (2018). Species specificity of type III interferon activity and development of a sensitive luciferase-based bioassay for quantitation of mouse interferon-lambda. J. Interferon Cytokine Res. 38 (11), 469–479. 10.1089/jir.2018.0066 30335553 PMC6249671

[B37] Kang J. A. Jeon Y. J. (2020). Emerging roles of USP18: from biology to pathophysiology. Int. J. Mol. Sci. 21 (18), 6825. 10.3390/ijms21186825 32957626 PMC7555095

[B38] Keser Y. Bumin Z. S. Perez Valiente A. Jacobs S. O. Boulant S. Stanifer M. L. (2026). Basal IFNlambda2/3 signaling is required for ISG expression and viral control in human intestinal epithelial cells. PLoS Pathog. 22 (1), e1013857. 10.1371/journal.ppat.1013857 41525418 PMC12822948

[B39] Kotenko S. V. Gallagher G. Baurin V. V. Lewis-Antes A. Shen M. Shah N. K. (2003). IFN-lambdas mediate antiviral protection through a distinct class II cytokine receptor complex. Nat. Immunol. 4 (1), 69–77. 10.1038/ni875 12483210

[B40] Lauber C. Vieyres G. Terczynska-Dyla E. Anggakusuma Dijkman R. Gad H. H. (2015). Transcriptome analysis reveals a classical interferon signature induced by IFNlambda4 in human primary cells. Genes. Immun. 16 (6), 414–421. 10.1038/gene.2015.23 26066369 PMC7308733

[B41] Lazear H. M. Schoggins J. W. Diamond M. S. (2019). Shared and distinct functions of type I and type III interferons. Immunity 50 (4), 907–923. 10.1016/j.immuni.2019.03.025 30995506 PMC6839410

[B42] Lex A. Gehlenborg N. Strobelt H. Vuillemot R. Pfister H. (2014). UpSet: visualization of intersecting sets. IEEE Trans. Vis. Comput. Graph 20 (12), 1983–1992. 10.1109/TVCG.2014.2346248 26356912 PMC4720993

[B43] Li B. Dewey C. N. (2011). RSEM: accurate transcript quantification from RNA-seq data with or without a reference genome. BMC Bioinforma. 12, 323. 10.1186/1471-2105-12-323 21816040 PMC3163565

[B44] Lin L. Wang X. Chen Z. Deng T. Yan Y. Dong W. (2023). TRIM21 restricts influenza A virus replication by ubiquitination-dependent degradation of M1. PLoS Pathog. 19 (6), e1011472. 10.1371/journal.ppat.1011472 37343022 PMC10325077

[B45] Love M. I. Huber W. Anders S. (2014). Moderated estimation of fold change and dispersion for RNA-seq data with DESeq2. Genome Biol. 15 (12), 550. 10.1186/s13059-014-0550-8 25516281 PMC4302049

[B46] Mesev E. V. LeDesma R. A. Ploss A. (2019). Decoding type I and III interferon signalling during viral infection. Nat. Microbiol. 4 (6), 914–924. 10.1038/s41564-019-0421-x 30936491 PMC6554024

[B47] Metz-Zumaran C. Uckeley Z. M. Doldan P. Muraca F. Keser Y. Lukas P. (2024). The population context is a driver of the heterogeneous response of epithelial cells to interferons. Mol. Syst. Biol. 20 (3), 242–275. 10.1038/s44320-024-00011-2 38273161 PMC10912784

[B48] Negishi H. Taniguchi T. Yanai H. (2018). The interferon (IFN) class of cytokines and the IFN Regulatory factor (IRF) transcription factor family. Cold Spring Harb. Perspect. Biol. 10 (11), a028423. 10.1101/cshperspect.a028423 28963109 PMC6211389

[B49] Park A. Iwasaki A. (2020). Type I and type III interferons - induction, signaling, evasion, and application to combat COVID-19. Cell Host Microbe 27 (6), 870–878. 10.1016/j.chom.2020.05.008 32464097 PMC7255347

[B50] Rock J. R. Hogan B. L. (2011). Epithelial progenitor cells in lung development, maintenance, repair, and disease. Annu. Rev. Cell Dev. Biol. 27, 493–512. 10.1146/annurev-cellbio-100109-104040 21639799

[B51] Schreiber G. (2017). The molecular basis for differential type I interferon signaling. J. Biol. Chem. 292 (18), 7285–7294. 10.1074/jbc.R116.774562 28289098 PMC5418031

[B52] Sheppard P. Kindsvogel W. Xu W. Henderson K. Schlutsmeyer S. Whitmore T. E. (2003). IL-28, IL-29 and their class II cytokine receptor IL-28R. Nat. Immunol. 4 (1), 63–68. 10.1038/ni873 12469119

[B53] Sun X. Li M. Dou Y. Wang Q. Lai J. Zhu J. (2025). Novel USP18 mutations lead to severe interferonopathy responsive to JAK inhibitor. Front. Immunol. 16, 1646996. 10.3389/fimmu.2025.1646996 40977694 PMC12443803

[B54] Tan K. S. Lim R. L. Liu J. Ong H. H. Tan V. J. Lim H. F. (2020). Respiratory viral infections in exacerbation of chronic airway inflammatory diseases: novel mechanisms and insights from the upper airway epithelium. Front. Cell Dev. Biol. 8, 99. 10.3389/fcell.2020.00099 32161756 PMC7052386

[B55] Tan K. S. Liu J. Andiappan A. K. Lew Z. Z. R. He T. T. Ong H. H. (2025). Unique immune and other responses of human nasal epithelial cells infected with H5N1 avian influenza virus compared to seasonal human influenza A and B viruses. Emerg. Microbes Infect. 14 (1), 2484330. 10.1080/22221751.2025.2484330 40126073 PMC11980200

[B57] Van Winkle J. A. Constant D. A. Li L. Nice T. J. (2020). Selective interferon responses of intestinal epithelial cells minimize tumor necrosis factor alpha cytotoxicity. J. Virol. 94 (21), e00603-20. 10.1128/JVI.00603-20 32847859 PMC7565621

[B58] Wang D. Y. Li Y. Yan Y. Li C. Shi L. (2015). Upper airway stem cells: understanding the nose and role for future cell therapy. Curr. Allergy Asthma Rep. 15 (1), 490. 10.1007/s11882-014-0490-0 25430951 PMC7088825

[B59] Wang Y. Guo L. Shi J. Li J. Wen Y. Gu G. (2023). Interferon stimulated immune profile changes in a humanized mouse model of HBV infection. Nat. Commun. 14 (1), 7393. 10.1038/s41467-023-43078-5 37968364 PMC10652013

[B60] Yan Y. Gordon W. M. Wang D. Y. (2013). Nasal epithelial repair and remodeling in physical injury, infection, and inflammatory diseases. Curr. Opin. Otolaryngol. Head. Neck Surg. 21 (3), 263–270. 10.1097/MOO.0b013e32835f80a0 23449287

[B61] Ye L. Schnepf D. Staeheli P. (2019). Interferon-lambda orchestrates innate and adaptive mucosal immune responses. Nat. Rev. Immunol. 19 (10), 614–625. 10.1038/s41577-019-0182-z 31201377

[B65] Zhang C. Lee H. Y. Shrivastava A. Wang R. McQuiston T. J. Challberg S. S. (2018). Long-term in vitro expansion of epithelial stem cells enabled by pharmocological inhibition of PAK1-ROCK-Myosin II and TGF-β signalling. Cell Rep. 25 (3), 598–610. 10.1016/j.celrep.2018.09.072 30332641 PMC6284236

[B62] Zhao X. Yu F. Li C. Li Y. Chao S. S. Loh W. S. (2012). The use of nasal epithelial stem/progenitor cells to produce functioning ciliated cells *in vitro* . Am. J. Rhinol. Allergy 26 (5), 345–350. 10.2500/ajra.2012.26.3794 22856354

[B63] Zhou Y. Zhou B. Pache L. Chang M. Khodabakhshi A. H. Tanaseichuk O. (2019). Metascape provides a biologist-oriented resource for the analysis of systems-level datasets. Nat. Commun. 10 (1), 1523. 10.1038/s41467-019-09234-6 30944313 PMC6447622

