# Supplementary material for: Differential host responses by the type I and III interferons in primary differentiated human nasal epithelial cells are a determinant of antiviral activity
Source: Front Pharmacol. 2026 Sep 15;17:1895412. doi: 10.3389/fphar.2026.1895412 (PMC13619389; doi:10.3389/fphar.2026.1895412)
Supplement: Supplementary file 1 [file Supplementaryfile1.zip › Supplementary Files/Supplementary Figures.docx]

Supplementary Material

**Supplementary Figure S1.** Raw images of differentiated hNEC cultures (main Figure 1A). Sections were stained with acetylated α-tubulin and MUC5AC antibodies or with the secondary antibodies only for background control.

**Supplementary Figure S2.** UpSet plot showing the number of intersecting genes from the top 5 groups with the greatest number of DEGs (IFN-β1 6h, IFN-β1 24h, IFN-λ1 72h, IFN-λ3 6h, and IFN-λ3 72h) in the apical-only IFN treatment route.

**Supplementary Figure S3.** Cell viability assay and expression of inflammatory response-related genes with increasing dose of IFN-β1 or IFN-λ1. **(A)** Cell viability of IFN treatment relative to vehicle control assessed by the LDH assay on basal media. Data is presented as mean ± SD (n=3); statistical significance was analysed by two-way ANOVA (compared to vehicle control, dotted line) with Dunnett's multiple comparisons test, n.s. not significant. **(B)** Relative expression of selected inflammatory response-related genes in IFN-treated, uninfected hNECs, measured by qPCR (n=3). MX1 served as an indicator of IFN stimulation.

**Supplementary Figure S4.** Gene expression of type I IFN receptors (IFNAR1 and IFNAR2) and type III IFN receptors (IFNLR1 and IL10RB) in vehicle control groups extracted from the RNA-sequencing data in this study. TPM: transcripts per million.

**Supplementary Methods**

**Lactate Dehydrogenase (LDH) Assay**

The hNECs were treated with 25 ng/ml, 250 ng/ml or 1000 ng/ml of IFN-β1 or IFN-λ1 at the basal-only or apical with basal route for 24h. Basal media was harvested and diluted 50X with LDH Storage Buffer (200mM Tris-HCl pH 7.3, 10% Glycerol, 1% BSA) and incubated 1:1 with LDH Detection Reagent (Promega) at room temperature for 1h with rocking. Luminescence was recorded with the Tecan M200 Pro plate reader. Relative cell viability to vehicle control was calculated with the following formula:

$$Relative cell viability= \frac{(vehicle control-medium background)}{(treatment-medium background)}$$

**Reverse Transcription Quantitative Polymerase Chain Reaction (RT-qPCR)**

The hNECs were treated with 25 ng/ml, 250 ng/ml or 1000 ng/ml of IFN-β1 or IFN-λ1 at the basal-only or apical with basal route for 24h. RNA was extracted with the Direct-zol RNA Miniprep Kit (Zymo Research) according to the manufacturer’s instructions. Reverse transcription was performed with the qScript Ultra SuperMix (Quantabio). Gene expression was analysed by qPCR with the GoTaq qPCR Master Mix (Promega) on the FMR3 qPCR machine (Vazyme). Relative gene expression was normalised to the PGK1 reference gene using the 2^-ΔΔCt^ method.

|  | **Forward Primer** | **Reverse Primer** |
| --- | --- | --- |
| **PGK1** | CTCAACAACATGGAGATTGG | CTTTGGACATTAGGTCTTTGAC |
| **MX1** | CAGGCTTTGTGAATTACAGG | TCTTCAATTTTGGACTTGGC |
| **CXCL13** | GCTTGAGGTGTAGATGTGTCC | CCCACGGGGCAAGATTTGAA |
| **GSDMB** | ATGTAGACTCAACGGGAGAGTT | GTAGCCAGATACTGCTGGGATA |
| **TNFRSF18** | CGTTCTCCAACACGACTTCATCC | ACGTGCAGACTGCATCCATGCT |
| **MEP1B** | CAGTGACTCTGATCTCCTAAAGTTGA | TGCACGAGTCCATAAAACTCA |
| **IDO1** | GCCAGCTTCGAGAAAGAGTTG | ATCCCAGAACTAGACGTGCAA |

| **Standard Cycling Conditions** | | | |
| --- | --- | --- | --- |
| **Step** | **Cycles** | **Temperature** | **Time** |
| GoTaq Hot Start Polymerase Activation | 1 | 95°C | 600s |
| Denaturation | 40 | 95°C | 15s |
| Annealing and Extension |  | 60°C | 60s |
| Melting | 1 | 95°C | 10s |
|  |  | 60°C | 60s |
|  |  | 97°C | 1s |
